# Multi-drug and β-lactam resistance in Escherichia coli and food-borne pathogens

**DOI:** 10.1093/eurpub/ckac131.572

**Published:** 2022-10-25

**Authors:** M Costa, M Cardo, P Soares, M Cara d'Anjo, A Leite

**Affiliations:** 1 NOVA-National School of Public Health, Public Health Research Centre, Universidade NOVA de Lisboa, Lisbon, Portugal; 2 Comprehensive Health Research Center, Universidade NOVA de Lisboa, Lisbon, Portugal; 3 Centre for Interdisciplinary Research in Animal Health, Faculty of Veterinary Medicine, University of Lisbon, Lisbon, Portugal; 4 Veterinary Public Health Department, Directorate-General of Food and Veterinary, Lisbon, Portugal

## Abstract

Antimicrobial resistance (AMR) is one of the top ten global public health threats facing humanity by menacing the effective use of available antimicrobials for treatment of an ever-increasing range of infections caused by bacteria. Its potential recognize the transmission to humans by contact with commensal and zoonotic resistant bacteria from animal and food sources. The aim of this study is to describe multi-drug resistance (MDR) profile in Campylobacter (n = 541), Salmonella (n = 919) and commensal Escherichia coli (n = 2777) isolated from animal and food samples between 2014 and 2019 in Portugal. Antimicrobial susceptibility testing results to fluoroquinolones/quinolones, macrolides, 3rd generation cephalosporins, polymyxins, carbapenems, penicillins, aminoglycosides, tetracyclines, sulphonamides, trimethoprim and chloramphenicol were clustered using k-modes. Clusters were described by population (broilers, broilers meat, turkeys, pigs, pig meat), AMR classification (mono/dual-resistance, MDR to 3-4, 5-6 and ≥7), β-lactamases production, sample stage (farm, slaughterhouse, processing plant, retail), season, and year. Overall, the results suggest an escalating MDR behavior from farm to post-farm stages in all bacteria, including E. coli MDR to critically important antimicrobials, and that Salmonella (fluoro)quinolones resistance may be associated with broilers. Most ESBL/AmpC producing E. coli were resistant to both C3G and C4G in isolates from animal and food samples (respectively, 77%; n = 846; and 74%; n = 188). The statistical method, K-modes, offers an overall visualization of the data with the identification of AMR profiles that have been further described using surveillance variables. Our results provide relevant information to support policy and decision making to tackle MDR in farm and post-farm stages.

**Key messages:**

• An escalating multi drug resistance behavior from farm to post-farm stages.

• Escherichia coli multi drug resistance to critically important antimicrobials.

